# Conceptual Reconsideration of the Classification of Pulmonary Hypertension: From 5 Groups to 2 Groups

**DOI:** 10.1016/j.jacasi.2026.02.015

**Published:** 2026-04-01

**Authors:** Yunshan Cao, Wenjie Dong, Yiwei Shi, Xuechun Sun, Hongling Su, Dili Xie, Xuebin Han, Yanqing Guo, Yuhu He, Fei Gao, Hailong Dai, Wei Huang, Hongyan Tian

**Affiliations:** a Heart, Lung and Vessels Center, Sichuan Provincial People’s Hospital, University of Electronic Science and Technology of China, Chengdu, Sichuan, China; b NHC Key Laboratory of Pneumoconiosis, Shanxi Key Laboratory of Respiratory, Department of Pulmonary and Critical Care Medicine, The First Hospital of Shanxi Medical University, The First Clinical Medical College of Shanxi Medical University, Taiyuan, Shanxi, China; c Department of Cardiology, The First People’s Hospital of Tianshui, Tianshui, Gansu, China; d Department of Cardiology, Pulmonary Vascular Disease Center, Gansu Provincial Hospital, Lanzhou, Gansu, China; e Cardiovascular Medicine, Shanxi Cardiovascular Hospital, Taiyuan, Shanxi, China; f Geriatric Medicine Center, The Fifth People’s Hospital of Shanxi Province, Taiyuan, Shanxi, China; g Department of Cardiology, The Second Xiangya Hospital, Central South University, Changsha, Hunan, China; h Pulmonary Vascular Diseases Center, Beijing Anzhen Hospital, Capital Medical University, Beijing, China; i Department of Cardiology, Key Laboratory of Cardiovascular Disease of Yunnan Province, Yan'an Affiliated Hospital of Kunming Medical University, Yunnan, Kunming, China; j Pulmonary Hypertension Diagnosis and Treatment Center, Department of Cardiovascular Medicine, Cardiovascular Research Center, The First Affiliated Hospital of Chongqing Medical University, Chongqing, China; k Department of Peripheral Vascular Diseases, The First Affiliated Hospital of Xi’an Jiaotong University, Xi'an, Shaanxi, China

**Keywords:** binary framework, diagnostic pathway, large-vessel stenosis, microvasculopathy, PH classification, pulmonary hypertension

## Abstract

Pulmonary hypertension (PH) is a hemodynamic condition arising from heterogeneous, often overlapping triggers. Although the etiology-driven 5-group classification provides an essential framework for disease definition and research standardization, it is frequently challenged in real-world practice, where cardiopulmonary multimorbidity is common and often delays therapeutic decision making. Here, we propose a complementary, treatment-oriented binary framework that stratifies pulmonary hypertension by dominant structural mechanisms: pulmonary microvasculopathy versus pulmonary large-vessel stenosis. Although anatomically grounded, this distinction primarily reflects pathophysiological processes, hemodynamic behaviors, and therapeutic responsiveness. Rather than supplanting current classifications, it highlights lesion-level features directly relevant to therapeutic strategy. We outline pragmatic diagnostic pathways integrating anatomical imaging, hemodynamic confirmation, and systematic evaluation of cardiopulmonary comorbidities, emphasizing early identification of potentially revascularizable large-vessel disease, where surgical or endovascular intervention may yield substantial and durable clinical benefit. This pathophysiology-driven perspective aims to simplify frontline decision making, reduce diagnostic delay, and support treatment precision across diverse clinical settings.

## Highlights

• Pulmonary hypertension is characterized by heterogeneous and often overlapping etiologies, complicating diagnosis and treatment in real-world clinical practice.
• We propose a complementary, treatment-oriented binary framework distinguishing pulmonary microvasculopathy from pulmonary large-vessel stenosis to guide clinical management.
• The review outlines pragmatic diagnostic pathways and mechanism-informed treatment strategies applicable across diverse pulmonary hypertension phenotypes.
• Future studies should validate the impact of this framework on patient-centered outcomes and health care resource utilization in global settings.

The current international clinical classification of pulmonary hypertension (PH), as updated in the 2022 European Society of Cardiology/European Respiratory Society guidelines and discussed at the Seventh World Symposium on Pulmonary Hypertension 2024, serves as the cornerstone of contemporary PH management. Developed through extensive evidence synthesis by expert task forces, this framework categorizes PH into 5 groups primarily on the basis of etiology, pathophysiology, and therapeutic considerations, providing essential guidance for etiologic identification and research standardization.^1^^,^^2^

However, PH is inherently heterogeneous in clinical phenotype. In real-world practice, substantial variability exists in patient characteristics, regional disease prevalence, and health care resources across different geographic settings. A recent interim analysis of the Pulmonary Hypertension Global Patient Survey underscored this imbalance, with 75% of respondents originating from Europe and North America while South America, Africa, Asia, and Oceania together accounted for only 25%. Moreover, nearly 80% of patients reported never having completed a patient-reported outcome measure.^3^ This geographic and data imbalance suggests that, despite their robustness, current guideline frameworks may incompletely capture the complexity of multimorbidity and region-specific disease patterns, including Takayasu arteritis–associated PH and fibrosing mediastinitis–associated PH (FM-PH), which are more prevalent in Asian populations.^4^, ^5^, ^6^, ^7^, ^8^, ^9^

Beyond serving specialists, a core objective of clinical classification is to facilitate knowledge translation to nonspecialists and support practical decision making across diverse clinical environments. In this context, the increasing burden of overlapping comorbidities, the risk of etiologic misclassification, and the need for streamlined, treatment-relevant workflows highlight the limitation of relying exclusively on etiology-based categorization. To address these challenges, we propose a conceptual reconsideration of PH classification through a complementary, pathophysiology-based binary framework designed to prioritize treatment-relevant structural pathology, optimize local health care resource utilization, and facilitate timely identification of potentially curable disease.

Importantly, this binary framework builds on the established 5-group classification and introduces a decision-oriented perspective that aligns anatomical pathology with therapeutic actionability.

## Current Classification of PH

**Figure 1:**
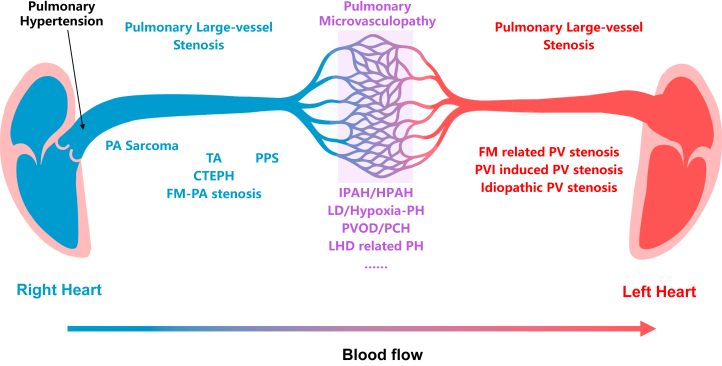
Diverse Etiologies of PH The figure illustrates the different PH cases along the pulmonary circulation. These cases can be classified into 3 major categories on the basis of the predominant site of obstruction: 1) pulmonary arterial large-vessel stenosis (blue), including CTEPH, PA sarcoma, and vasculitis; 2) pulmonary microvasculopathy (purple), encompassing PAH and related small-vessel disorders such as PVOD; and 3) pulmonary venous large-vessel stenosis (red), such as fibrosing mediastinitis–associated PV stenosis and stenosis induced by pulmonary vein isolation. CTEPH = chronic thromboembolic pulmonary hypertension; FM = fibrosing mediastinitis; HPAH = heritable pulmonary arterial hypertension; IPAH = idiopathic pulmonary arterial hypertension; LD = lung disease; LHD = left heart disease; PA= pulmonary artery; PAH = pulmonary arterial hypertension; PCH = pulmonary capillary hemangiomatosis; PH = pulmonary hypertension; PPS = peripheral pulmonary artery stenosis; PV = pulmonary vein; PVI = pulmonary vein isolation; PVOD = pulmonary veno-occlusive disease; TA = Takayasu arteritis.

PH represents an abnormal hemodynamic state, defined by the 2022 European Society of Cardiology/European Respiratory Society guidelines as a resting mean pulmonary arterial pressure >20 mm Hg. PH is not a single disease entity, but rather a heterogeneous clinical syndrome encompassing a wide spectrum of underlying diseases and pathophysiological mechanisms. These include pulmonary arterial hypertension (PAH); PH associated with left heart disease, lung diseases, and/or hypoxia; chronic thromboembolic pulmonary disease; as well as pulmonary vascular obstructions such as pulmonary artery stenosis and pulmonary vein stenosis (Figure 1).^1^

The clinical classification of PH was first introduced at the World Health Organization (WHO) symposium in Geneva in 1973, at which time PH was broadly categorized into primary PH and secondary PH. As understanding of the etiology and pathobiology of pulmonary vascular disease has advanced, this classification scheme has undergone multiple iterations and refinement. The contemporary WHO classification was formally established at the Evian workshop in 1998 and subsequently refined through a series of international consensus meetings held in Venice (2003), Dana Point (2008), and Nice (2013, 2018, 2022).^10^, ^11^, ^12^ On the basis of etiologic considerations, the current WHO classification stratifies PH into 5 groups: group 1, PAH; group 2, PH associated with left heart disease; group 3, PH associated with lung diseases and/or hypoxia; group 4, PH associated with pulmonary artery obstructions; and group 5, PH with unclear and/or multifactorial mechanisms.^1^

## Limitations and Clinical Challenges of the Current Classification System

Although the 5-group classification has been instrumental in advancing the field of PH, its strong emphasis on etiology presents substantial challenges in contemporary clinical practice. In real-world settings, many patients do not exhibit a single, clearly dominant cause of PH. Instead, multiple coexisting etiologies are common, and pathophysiological mechanisms frequently overlap across classification groups, complicating strict single-category assignment. Registry data suggest that group 1 PAH and group 2 PH associated with left heart disease often share key mechanistic features, including endothelial dysfunction, inflammation-driven vascular remodeling, and impaired pulmonary vascular compliance.^13^^,^^14^ These shared pathways blur traditional etiologic boundaries and make categorical classification inherently ambiguous. Accordingly, what is labeled as “etiology” in the current framework is often better conceptualized as a constellation of triggering or contributory factors—such as genetic susceptibility, increased pulmonary blood flow, retrograde transmission of elevated left-sided filling pressures, systemic inflammation, vasoconstriction or vasospasm, and pulmonary vascular stenosis—rather than a single causative mechanism.^8^^,^^15^, ^16^, ^17^

The high prevalence of cardiopulmonary comorbidities further challenges the applicability of traditional PH classification systems. Large clinical registries indicate that 60% to 85% of patients with PH have significant cardiopulmonary comorbidities; even in rigorously screened pivotal clinical trials, approximately 50% of participants exhibit such overlap.^2^ In Pulmonary Vascular Disease Phenomics (PVDOMICS) cohort, 57% of patients initially classified as group 2 PH also fulfilled criteria for a group 2 and 3 overlap phenotype.^18^ Similarly, the Atherosclerosis Risk in Communities (ARIC) study reported that approximately 75% of patients with cardiovascular disease had concomitant chronic obstructive pulmonary disease (COPD) or restrictive lung disease.^19^ In these cohorts, substantial proportions of patients assigned to a given PH group exhibit features of other categories, reflecting frequent cardiopulmonary multimorbidity and overlapping pathophysiology. Clustering analyses further expose the limitations of purely etiologic classifications. In the COMPERA (Comparative, Prospective Registry of Newly Initiated Therapies for Pulmonary Hypertension) registry, only 12.6% of patients labeled as idiopathic PAH displayed a “classic” PAH phenotype whereas many showed predominant left heart– or lung-related features.^20^ Similar overlap has been reported in mechanistic cohorts in chronic thromboembolic pulmonary hypertension (CTEPH). Hemodynamically, elevated left ventricular filling pressures are reported in 11% to 37% of patients, indicating frequent left heart overlap.^21^ Clinically, large-scale registries across North America, Europe, and Asia reveal a substantial burden of respiratory comorbidities, with COPD and interstitial lung disease affecting 4% to 34% of cohorts, alongside significant prevalences of systemic disorders.^22^, ^23^, ^24^, ^25^ Beyond CTEPH, heterogeneity is also pronounced in less common PH subtypes, including Takayasu arteritis–associated PH and FM-PH.^26^^,^^27^

Collectively, these observations underscore a central challenge of the current classification system: real-world populations with PH often do not conform to a single dominant etiologic category, increasing the risk of misclassification and delayed recognition of alternative or potentially treatable disease substrates.

## Pathophysiology-Based Binary Framework of PH

Although the triggers of PH are traditionally grouped into 5 etiologic categories, the pathophysiological processes that ultimately lead to elevated pressure in main pulmonary artery converge on 2 core structural mechanisms: *pulmonary microvasculopathy* (PMV) and *pulmonary large-vessel stenosis* (PLVS). Importantly, these 2 mechanisms are not alternative etiologies but represent distinct final common pathways with different therapeutic implications. PH driven by PLVS is characterized by focal or segmental narrowing of large pulmonary arteries or veins and is often amenable to surgical or interventional treatment, including pulmonary endarterectomy, balloon angioplasty, or stent implantation. Such approaches can achieve substantial hemodynamic improvement and, in selected patients, near-curative outcomes.^28^, ^29^, ^30^ In contrast, PH caused by PMV primarily affects the distal pulmonary microcirculation and is managed predominantly with pharmacological therapies targeting the underlying pathogenic pathways. Although targeted therapies have significantly improved clinical outcomes, PMV remains a major therapeutic challenge.^31^^,^^32^ To address the clinical complexity and etiologic overlap inherent in the traditional 5-group classification, we therefore propose a simplified, pathophysiology-based binary framework centered on these 2 fundamental mechanisms (Central Illustration).

**Central Illustration:**
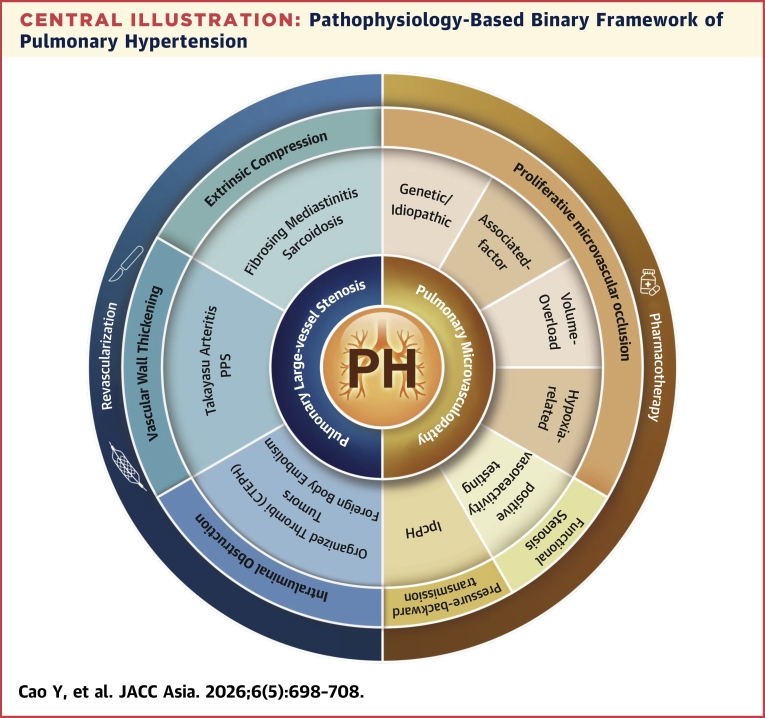
Pathophysiology-Based Binary Framework of Pulmonary Hypertension PH is categorized according to 2 major pathophysiological mechanisms: pulmonary large-vessel stenosis (potentially revascularizable) and pulmonary microvasculopathy (predominantly pharmacotherapy-focused), with specific etiologies and treatment strategies corresponding to each category. CTEPH = chronic thromboembolic pulmonary hypertension; IpcPH = isolated postcapillary pulmonary hypertension; PH = pulmonary hypertension; PPS = peripheral pulmonary artery stenosis.

## Pulmonary Microvasculopathy

PMV is defined by abnormal proliferative and functional changes involving distal pulmonary arterioles, venules, and capillaries, resulting in progressive luminal narrowing and increased pulmonary vascular resistance. Mechanistically, PMV can be subdivided into 3 major patterns: proliferative microvascular occlusion, vasoreactive dysfunction, and pressure-backward transmission.^32^, ^33^, ^34^

*Proliferative microvascular occlusion* represents a fixed structural form of microvascular disease. It is characterized by endothelial dysfunction with excessive cellular proliferation and fibrotic remodeling. Histopathological features include endothelial hyperplasia, smooth muscle cell proliferation, medial and intimal thickening with fibrosis, and muscularization of distal pulmonary vessels.^35^ Based on predominant triggers, this pattern may manifest as several subtypes: a *genetic or idiopathic type*, arising from inherited susceptibility or unidentified causes; an *associated-factor type*, secondary to drugs, toxins, connective-tissue diseases, or systemic conditions; a *volume-overload type*, observed in high-flow states such as systemic-to-pulmonary shunt congenital heart disease, single-lung physiology, or segmental pulmonary artery stenosis, in which sustained excessive flow and shear stress drive maladaptive microvascular remodeling^1^^,^^36^^,^^37^; and a *hypoxia-related type*, commonly associated with COPD, interstitial lung disease, or high-altitude exposure, where sustained hypoxia promotes vasoconstriction, inflammation, and vascular wall remodeling.^38^ Overlap among these triggers is frequently encountered in clinical practice.^2^^,^^39^ The *vasoreactive type* of PMV is dominated by reversible pulmonary vasoconstriction and is typified clinically by patients who demonstrate a positive response to acute pulmonary vasoreactivity testing.^40^ *Pressure-backward transmission* is primarily driven by left heart disease. In this pattern, elevated left atrial pressure is transmitted retrogradely through the pulmonary veins, initially producing passive pulmonary congestion and corresponding clinically to isolated postcapillary PH.^1^^,^^14^ With prolonged exposure, secondary structural changes—such as medial hypertrophy of distal pulmonary arteries and intimal fibrosis—may develop, leading to a combined pre- and postcapillary phenotype.^41^

## Pulmonary Large-Vessel Stenosis

PLVS is unified by physical narrowing or occlusion of large pulmonary arteries or veins, leading to elevated pulmonary arterial pressure. This category primarily encompasses entities within WHO group 4, selected subtypes of group 5 (notably fibrosing mediastinitis), and pulmonary venous stenosis.^4^^,^^5^^,^^42^ On the basis of the mechanisms of luminal compromise, PLVS can be subdivided into 3 patterns: intraluminal obstruction, vascular wall thickening, and extrinsic compression. *Intraluminal obstruction* is exemplified by CTEPH, in which incompletely resolved thrombi undergo organization and fibrosis, leading to fixed luminal occlusion.^42^, ^43^, ^44^ Tumor emboli or foreign-body emboli may produce similar effects.^45^ *Vascular wall thickening* is characteristic of inflammatory vasculopathies, such as Takayasu arteritis, where chronic inflammation induces fibrotic mural thickening and progressive luminal narrowing.^29^^,^^46^, ^47^, ^48^ *Extrinsic compression* arises from structures external to the vessel, including fibrosing mediastinitis and mediastinal tumors, which mechanically compress pulmonary arteries or veins.^4^^,^^49^ Notably, persistent hemodynamic stress in PLVS can also induce secondary distal microvasculopathy, resulting in composite phenotypes characterized by concomitant large-vessel stenosis and microvascular remodeling.^44^

## Diagnostic Pathway Based on the Binary Framework

In current clinical practice, the diagnostic evaluation of PH remains largely epidemiology- and etiology-driven, with initial efforts focused on identifying PH associated with left heart disease (group 2) or lung disease (group 3).^1^ Although this stepwise exclusion strategy is effective in patients with a single dominant etiology, it is often inadequate in those with complex cardiopulmonary comorbidities, substantially increasing the risk of misdiagnosis and diagnostic delay. Because PLVS frequently coexists with left heart or lung disease, these revascularizable lesions are often obscured in traditional workflows, leading to their sole classification into other PH groups. For example, patients with left heart disease and concomitant chronic thromboembolic pulmonary disease may be classified as group 2 PH whereas those with fibrosing mediastinitis and COPD may be labeled group 3, thereby delaying referral for potentially curative surgical or endovascular interventions.^50^^,^^51^ Respiratory comorbidities further confound symptom interpretation and imaging findings, obscuring proximal pulmonary vascular disease. These diagnostic challenges are reflected in substantial real-world delays. CTEPH is frequently misdiagnosed as asthma, COPD, or PAH, with reported diagnostic delays ranging from 14 months to 3.9 years^39^^,^^50^^,^^52^, ^53^, ^54^; FM-PH often lacks distinctive clinical features, with diagnostic delays of up to 3 years reported in a French referral cohort.^51^ Similarly, in Takayasu arteritis–associated pulmonary artery stenosis, a Chinese multicenter study documented a median diagnostic delay of 2.0 years from symptom onset.^5^

### Introduction of the stenosis-PH-comorbidity workflow

**Figure 2:**
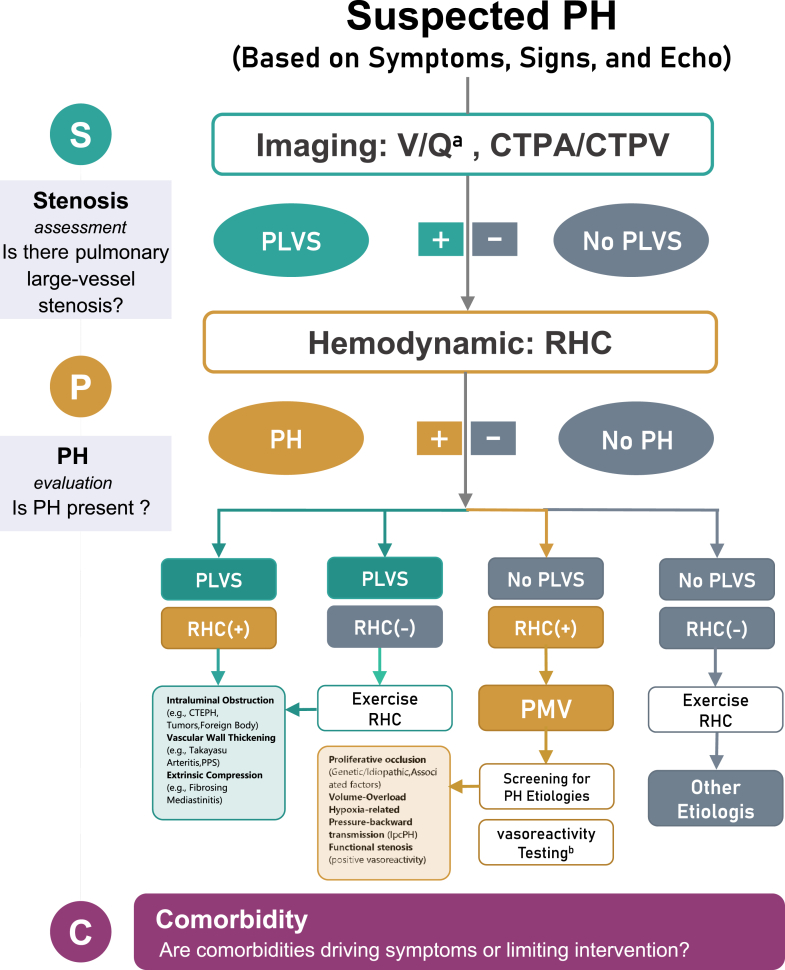
Diagnostic Pathway Based on the Binary Framework The figure illustrates the “SPC” (stenosis-PH-comorbidity) strategy, which stratifies diagnosis into 3 domains. (S) Stenosis assessment: Noninvasive imaging (typically V/Q scan and/or CTPA/V) serves as the filter to screen for PLVS. (P) PH evaluation: RHC defines the definitive hemodynamic status. (C) Comorbidity assessment: Concurrent assessment determines whether comorbidities are contributing to symptoms or limiting therapeutic interventions. ^a^V/Q scanning is well established for CTEPH but may be insensitive to other forms of pulmonary large-vessel stenosis; a negative result should not preclude pulmonary computed tomography angiography when clinical suspicion persists. ^b^Indicated for IPAH, HPAH, or drug-induced PAH according to the current PH guideline. CTPA/V = computed tomography pulmonary angiography/venography; IpcPH = isolated postcapillary pulmonary hypertension; PLVS = pulmonary large-vessel stenosis; PMV = pulmonary microvasculopathy; RHC = right heart catheterization; V/Q = ventilation/perfusion; other abbreviations as in Figure 1.

In light of these realities, we advocate for the “SPC” (stenosis-PH-comorbidity) workflow based on a pathophysiology-based, lesion-oriented, binary framework. As illustrated in Figure 2, this approach integrates anatomical and hemodynamic evaluations to distinguish revascularizable pulmonary large-vessel disease from diffuse PMV, thereby reducing misclassification of patients within the potentially curable subset.

To operationalize this framework, the initial evaluation of patients with suspected PH should center on 2 pivotal investigations. First, noninvasive imaging is performed to screen for PLVS, with ventilation-perfusion (V/Q) scintigraphy and/or computed tomography pulmonary angiography with venography serving as the primary modalities. Second, right heart catheterization (RHC) is used to define the hemodynamic profile and confirm the presence of PH.^55^^,^^56^ Together, these complementary assessments provide a binary stenotic-hemodynamic filter that enables early identification of revascularizable disease. Nevertheless, clinicians should remain vigilant regarding the limitations of V/Q scanning. The current evidence base for V/Q imaging is derived predominantly from studies in CTEPH, whereas its diagnostic performance in other forms of PLVS is less well established and requires further validation.^55^ Importantly, in patients with a high clinical suspicion of PLVS—such as those presenting with the fibrosing mediastinitis dyad or triad or those demonstrating mosaic perfusion on noncontrast chest computed tomography—additional vascular imaging is warranted.^4^ In those scenarios, computed tomography pulmonary angiography/venography should be performed even in the setting of a negative V/Q scan and may reasonably be considered as a first-line modality, given its ability to directly visualize focal, segmental, or venous-side lesions that may be missed by perfusion-based screening. Finally, comprehensive assessment of cardiopulmonary comorbidities should be integrated throughout the diagnostic pathway. Comorbid conditions not only modulate symptom burden and prognosis but also critically influence therapeutic feasibility and strategy selection within the SPC framework.

On the basis of the intersection of anatomic findings and hemodynamic status, 4 distinct clinical scenarios emerge.

#### Scenario 1: Stenosis-positive/PH-positive

This phenotype represents confirmed PLVS with hemodynamically established PH and defines the prototypical revascularizable disease subset. Further etiologic investigation is also essential. Etiologically, this large-vessel stenosis is mainly driven by 3 distinct mechanisms: intraluminal obstruction (most notably organized thrombi in CTEPH, but also tumors or foreign bodies), vascular wall thickening (eg, Takayasu arteritis and peripheral pulmonary artery stenosis), or extrinsic compression (eg, fibrosing mediastinitis and sarcoidosis). Patients in this category are most likely to benefit from lesion-directed interventions, including endovascular revascularization or surgical correction, depending on lesion location, extent, morphology, and comorbidities. Early identification is critical, as timely restoration of pulmonary blood flow may halt or reverse downstream right ventricular remodeling and improve long-term outcomes. Clinicians should remain vigilant for concomitant PMV, as accumulating evidence indicates that many patients with chronic thromboembolic pulmonary disease or arteritis-related pulmonary artery stenosis have significant distal small-vessel involvement.^5^^,^^42^

#### Scenario 2: Stenosis-positive/PH-negative

This pattern suggests evident large-vessel stenosis in the absence of resting PH. Such patients may represent an early or compensated disease stage, in which limited lesion burden preserves resting pulmonary pressures and right heart function. In this context, exercise RHC may be considered to unmask occult hemodynamic impairment. Close surveillance is warranted, as this subgroup remains at risk for progression to overt PH and may benefit from pre-emptive or staged intervention in selected cases.

#### Scenario 3: Stenosis-negative/PH-positive

In the absence of revascularizable large-vessel lesions, this phenotype is more consistent with PMV. Management should therefore shift toward systematic etiologic evaluation, including assessment for PAH and PH associated with left heart disease, lung disease, or systemic disorders. Acute vasodilator testing is typically prioritized in selected cases.

#### Scenario 4: Stenosis-negative/PH-negative

This scenario effectively excludes both PLVS and resting PH, redirecting clinical attention toward alternative cardiopulmonary or systemic contributors to symptoms, such as deconditioning, parenchymal lung disease, left-sided cardiac abnormalities, or extracardiopulmonary causes. Importantly, exercise-induced PH remains a consideration, particularly in symptomatic patients with preserved resting hemodynamics. In selected cases, exercise RHC may be warranted to unmask occult hemodynamic abnormalities. Once both resting and exercise-related pulmonary vascular pathology are reasonably excluded, management should focus on targeted evaluation and treatment of identified comorbidities.

### Conceptual significance

Together, these 4 scenarios illustrate how the SPC framework reframes PH evaluation from a purely etiology-based classification toward a lesion-level, decision-oriented diagnostic process. Designed to complement existing classifications, this framework targets the critical diagnostic window preceding reliable etiologic attribution. By anchoring early diagnostic reasoning at the intersection of anatomy and hemodynamic profiles, the SPC framework prioritizes a single actionable question: Is there a large-vessel stenosis amenable to revascularization? Answering this question early prevents premature commitment to medical management in patients who might benefit from intervention. In this context, SPC functions as a treatment-decisive triage gate that structures the diagnostic workflow. This hierarchical approach minimizes diagnostic ambiguity, limits phenotypic misclassification, and ensures that potentially curable large-vessel substrates are not overlooked. Finally, embedding comorbidity assessment as a parallel axis—rather than a terminal exclusion step—aligns SPC with real-world complexity, where comorbidities shape symptom burden, procedural risk, and therapeutic feasibility across both PLVS and microvasculopathy phenotypes.

## Therapeutic Strategies Based on the Binary Framework

**Figure 3:**
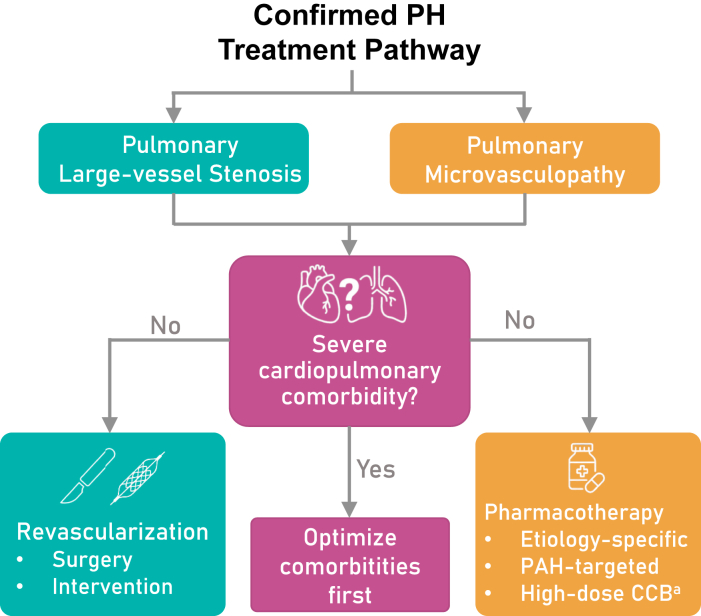
Therapeutic Pathway Based on the Binary Framework The figure outlines the treatment decision-making process for PH, distinguishing between pulmonary large-vessel stenosis and pulmonary microvasculopathy. An important modifying factor in this process is the presence of severe cardiopulmonary comorbidities. If present, therapeutic efforts should prioritize optimization of comorbid conditions. In their absence, patients are directed toward mechanism-specific strategies: revascularization for large-vessel stenosis or pharmacological therapy for microvascular involvement, including etiology-specific treatments, PAH-targeted therapies, and high-dose CCB therapies in appropriately selected patients. ^a^Restricted to patients with a positive acute vasoreactivity test. CCB = calcium-channel blocker; other abbreviations as in Figure 1.

PH management encompasses supportive care, etiologic treatment, and comprehensive comorbidity management. However, targeted intervention addressing the dominant pulmonary vascular mechanism remains the cornerstone of therapy (Figure 3).

Within the SPC workflow, the binary framework does not serve as a therapeutic algorithm itself, but rather as a mechanism-informed decision tool that guides selection of appropriate treatment pathways. In patients where PH is driven predominantly by PMV, subsequent management should proceed along established etiology-based pathways, with treatment stratified by cardiopulmonary comorbidity burden. In patients without significant cardiopulmonary comorbidities whose clinical phenotype and hemodynamic profile are consistent with precapillary PAH, PAH-targeted therapies should be initiated and escalated in accordance with current guidelines. Among these patients, vasoreactivity testing may identify a small subset who benefit from high-dose calcium-channel blocker therapy. Conversely, in patients with left heart disease, chronic lung disease, or systemic disorders, treatment should prioritize optimization of the primary condition and PAH-targeted therapies should not be empirically applied without a clear mechanistic rationale. In contrast, in patients identified as having anatomically discrete pulmonary arterial or venous stenosis, lesion-directed surgical or interventional therapy constitutes the cornerstone of management. Therapeutic planning in this group should be multidisciplinary and integrate symptom burden, lesion anatomy and morphology, severity of obstruction and downstream perfusion impairment, baseline hemodynamic status and right ventricular function, procedural risk, and comorbidities. Importantly, the presence of PH should be regarded as one manifestation along a disease continuum rather than the sole determinant of intervention, emphasizing individualized risk-benefit assessment.

Importantly, PLVS and PMV frequently coexist. Chronic proximal obstruction may be accompanied by distal small-vessel remodeling, and established microvasculopathy may limit the hemodynamic reversibility achievable by revascularization. At present, there is no validated approach to directly quantify the microvascular disease burden in conditions dominated by large-vessel lesions; therefore, assessment remains indirect and relies on the overall hemodynamic profile, the distribution/extent of proximal disease, and the clinical response after lesion-directed therapy. In such mixed phenotypes, a pragmatic strategy is to prioritize evaluation for revascularizable lesions while anticipating that a subset of patients may require adjunctive, mechanism-concordant medical therapy to address a residual microvascular component.

## Conclusions

PH is a hemodynamic condition arising from heterogeneous etiologies but converging on a limited number of core pathophysiological mechanisms. Although the current 5-group classification, which is fundamentally etiology-based, remains essential for etiologic attribution and research standardization, it often provides limited guidance for early diagnostic prioritization and treatment selection in patients with complex comorbidities or overlapping disease substrates.

The pathophysiology-based binary framework that distinguishes PLVS from PMV offers a pragmatic, lesion-oriented, treatment-decisive complement to existing classifications. By prioritizing identification of potentially revascularizable large-vessel disease before downstream etiologic labeling, the SPC framework may reduce diagnostic delay and improve mechanism-informed clinical decision making.

Given the frequent coexistence of microvascular and large-vessel mechanisms, the binary framework is not designed as a rigid categorization but as a guide for individualized, mechanism-informed management. Future prospective studies incorporating patient-reported outcome measures and health-related quality of life measures will be critical to evaluate the real-world impact of this framework on survival, quality of life, and health care resource utilization.

## Funding Support and Author Disclosures

This work was supported by the National Natural Science Foundation of China (no. U25A2001), and the Natural Science Foundation of Gansu Province (no. 24JRRE012). The authors have reported that they have no relationships relevant to the contents of this paper to disclose.
